# Phosphorylation of the C-terminal tail of proteasome subunit α7 is required for binding of the proteasome quality control factor Ecm29

**DOI:** 10.1038/srep27873

**Published:** 2016-06-15

**Authors:** Prashant S. Wani, Anjana Suppahia, Xavier Capalla, Alex Ondracek, Jeroen Roelofs

**Affiliations:** 1 Graduate Biochemistry Group, Department of Biochemistry and Molecular Biophysics, Kansas State University, 336 Ackert Hall, Manhattan, Kansas 66506, USA; 2 Molecular, Cellular and Developmental Biology Program, Division of Biology, Kansas State University, 338 Ackert Hall, Manhattan, Kansas 66506, USA

## Abstract

The proteasome degrades many short-lived proteins that are labeled with an ubiquitin chain. The identification of phosphorylation sites on the proteasome subunits suggests that degradation of these substrates can also be regulated at the proteasome. In yeast and humans, the unstructured C-terminal region of α7 contains an acidic patch with serine residues that are phosphorylated. Although these were identified more than a decade ago, the molecular implications of α7 phosphorylation have remained unknown. Here, we showed that yeast Ecm29, a protein involved in proteasome quality control, requires the phosphorylated tail of α7 for its association with proteasomes. This is the first example of proteasome phosphorylation dependent binding of a proteasome regulatory factor. Ecm29 is known to inhibit proteasomes and is often found enriched on mutant proteasomes. We showed that the ability of Ecm29 to bind to mutant proteasomes requires the α7 tail binding site, besides a previously characterized Rpt5 binding site. The need for these two binding sites, which are on different proteasome subcomplexes, explains the specificity of Ecm29 for proteasome holoenzymes. We propose that alterations in the relative position of these two sites in different conformations of the proteasome provides Ecm29 the ability to preferentially bind specific proteasome conformations.

The ubiquitin proteasome system (UPS) is present in all eukaryotes and enables cells to degrade many proteins in a highly regulated fashion^1^. Numerous enzymes are required to specifically label proteins that are destined for degradation with ubiquitin. Ubiquitin, a short polypeptide, is normally covalently attached to lysine residues present in the target protein. The ubiquitinated proteins become substrates for the proteasome, a 2.5 MDa protease complex. The proteasome holoenzyme consists of 33 unique polypeptides that are assembled into two main subcomplexes: the regulatory particle (RP) and the core particle (CP). The RP recognizes ubiquitinated substrates either through direct interaction with proteasome subunits that function as ubiquitin receptors, like Rpn10, Rpn13, or Rpn1, or through recognition of adaptors that bind ubiquitinated substrates and the proteasome, such as Rad23 or Dsk2^2^,^3^. The RP removes the ubiquitin from substrates and unfolds them. The unfolding occurs at an AAA-ATPase ring formed by the six homologous proteasome subunits Rpt1 to Rpt6. The Rpt ring abuts the CP and is responsible for threading substrates into the CP where the proteolytic active sites are located. The CP is formed by four hetero-heptameric rings that are stacked upon one another, resulting in a hollow, cylinder-like structure. CP subunits are of two types, α and β, arranged to form an α_1–7_β_1–7_β_1–7_α_1–7_ structure. β1, β2, and β5 are the catalytic subunits that provide the protease activity required for protein degradation.

The proteasome is an abundant complex in the cell, therefore it is important for the cell to assemble all 66 subunits that comprise this RP_2_-CP complex efficiently and correctly. To achieve this, proteasome assembly is tightly orchestrated in the cell with the help of ten dedicated assembly chaperones^4^,^5^. Five of these chaperones, Hsm3, Nas2, Nas6, Rpn14, and Adc17, are dedicated to RP assembly. The other five chaperones, Pba1, Pba2, Pba3, Pba4, and Ump1, assist in CP assembly. Interestingly, these chaperones not only promote the formation of specific subcomplexes, but several also prevent the premature association of RP and CP. For example, the CP chaperones Pba1-Pba2 prevent the association of RP with immature CP^6^,^7^. Similarly, the RP chaperones are capable of blocking mature CP from interacting with RP^8^,^9^,^10^,^11^. Thus, it appears that the final step in assembly, the association of CP with RP, is tightly controlled.

The chaperones, under normal conditions, only bind to proteasome subcomplexes and are not found on the holoenzyme or mature CP. Several other proteasome-associated components have been identified that have binding sites on either CP or RP^12^. However, these have also been found associated with 26S proteasomes or mature CP, suggesting they regulate proteasome activity or assist in proteasome function. Indeed, several assist in delivery of substrates to the proteasome, modify the ubiquitin chains on substrates, or change the hydrolytic activity of the core particle^12^. Ecm29 is a unique proteasome component as it is the only one known to bind to the RP (Rpt5) as well as the CP^13^,^14^. Nevertheless, it is normally only found on singly and doubly capped proteasomes, i.e. RP-CP complexes and RP_2_-CP complexes^13^,^15^.

Ecm29 is a large protein (210 kDa) predicted to contain 29 HEAT repeats. Both CryoEM analyses and structure predictions of Ecm29 suggest that Ecm29 forms an elongated and curved protein similar to many other proteins with multiple HEAT repeats^13^,^16^. Several functions have been proposed for yeast Ecm29 and the human ortholog KIAA0368. For example, it has been suggested to remodel proteasomes under stress conditions^17^,^18^, as well as play a role in localizing proteasomes to membrane components^19^,^20^,^21^, or movement of proteasomes in neurons^22^. Initially, it was thought to positively regulate proteasome function as it stabilizes the RP-CP interaction in the absence of nucleotide^13^,^15^. However, Ecm29 has been shown to inhibit proteasome activity *in vivo* and *in vitro*^14^,^23^.

The inhibition by Ecm29 involves two mechanisms: first, Ecm29 binding induces closure of the substrate entry channel^17^,^23^. This channel is found on the top and bottom of CP and is normally opened by RP. Second, Ecm29 reduces the ATPase activity of proteasomes. This ATPase activity is required to unfold substrates so that they can enter the CP^14^. Interestingly, Ecm29 has been found enriched on a variety of proteasome mutants^17^,^23^,^24^,^25^. These and other data led to the proposed function of Ecm29 as a quality control factor that recognizes aberrant proteasomes^14^,^23^,^24^,^25^.

It remains poorly understood why Ecm29 is enriched on mutant proteasomes. Ecm29 could specifically recognize faulty proteasomes^14^, it could be an unstable proteasome substrate that is stabilized resulting from reduced proteasome activity^24^,^25^, or it could be upregulated in strains with proteasome mutations^17^. To better understand how Ecm29 associates with the proteasome and regulates proteasome activity, it is crucial to understand where Ecm29 binds to the proteasome complex. Using a crosslinking approach, we previously identified the RP subunit Rpt5 as an interaction partner of Ecm29^14^. However, no CP subunits were identified.

Here, we report the identification of an Ecm29 binding site on the core particle subunit α7. Truncation of the C-terminal tail of α7 substantially diminishes the Ecm29 interaction with the proteasome in both wild type and in mutant proteasomes. Phenotypic analyses confirm that this tail of α7 is important for Ecm29 binding to proteasomes *in vivo* as well. Interestingly, the tail of α7 has a conserved acidic region that contains three serine residues that are known to be phosphorylated. Mutation of these sites indicates that Ecm29 depends on α7 phosphorylation for its interaction with the CP. Thus, our data reveal an additional level of complexity to the binding of Ecm29 to proteasomes and identify a function for the phosphorylation sites in the tail of α7.

## Results

### α7 subunit of CP as putative binding partner for Ecm29

**Figure 1:**
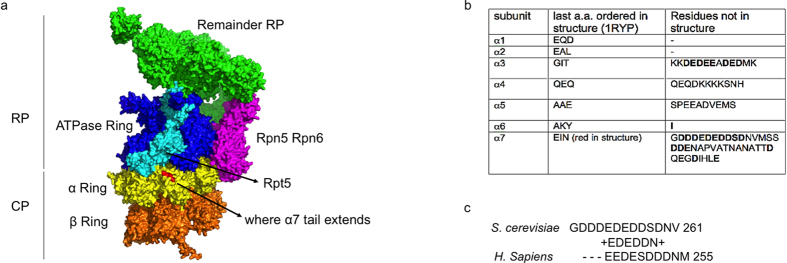
α7 as the putative binding site for Ecm29. **(a)** CryoEM-based model of the Proteasome^29^ (PDB- 4B4T) showing regulatory particle (RP) and core particle (CP). Rpt5 is displayed in cyan and other Rpt subunits in blue. Lid subunits Rpn5 and Rpn6 that interact with the CP are shown in purple and the remainder of RP is shown in green. The core particle α subunits are colored in yellow, with the last three ordered amino acids of α7 shwon in red. β subunits are colored in orange. **(b)** Amino acids sequences from the α-ring subunits beyond crystal structure^30^ and model presented^29^. Second column shows the last three ordered amino acids for each α subunit. Third column shows C-terminal amino acids that were not resolved in the structure. Consecutive acidic residues in C-terminal of α7 residues that forms the acidic patch are underlined as red line. **(c)** Sequence alignment of acidic patch in the C-terminal tail of α7 from *S. cerevisiae* and *H. sapiens*.

Ecm29 is predicted to bind CP as well as RP^13^. Based on the large size and predicted elongated and curved structure^13^,^16^, we hypothesized Ecm29 binds CP in close proximity to RP binding site Rpt5^14^. Recent mapping of the RP-CP interaction and structural insights from several cryoEM structures of the proteasome provide a detailed understanding of the location of Rpt5 with respect to CP^26^,^27^,^28^,^29^ (Fig. 1a). The C-terminus of Rpt5 docks into a pocket formed at the interface of the CP subunits α5 and α6^26^,^27^,^28^,^29^ and has also been shown to be able to occupy the pocket between α6 and α7^26^. The face of the CP cylinder provides the principal interaction surface with the RP. Here, the α-subunits have N-termini that extend and contribute to the formation of the CP gate^30^. This area is unlikely to be available for Ecm29 binding, because it is covered by the RP in the 26S proteasomes^27^,^28^,^29^. Thus, Ecm29 is more likely to bind to the lateral surfaces of α-subunits. Interestingly, four out of seven α-subunits (α3, α4, α5, and α7) have C-terminal amino acid sequences (≥10 residues) that are not resolved in the crystal structure of the CP and are likely to extend from the lateral surface of the CP^30^. Of these, α7 contains the longest extension, 40 residues, and this tail contains a patch of acidic amino acids (Fig. 1b). Human α7 has a similar C-terminal tail that extends beyond the crystal structure and harbors numerous acidic residues^31^ (Fig. 1c). Thus, features of the α7C-terminal tail are conserved from yeast to human. Since Ecm29 is conserved, presumably the binding site is as well. Figure 1a shows the last three resolved α7 residues in a 26S model in red. From this it is clear that the extended tail is in close proximity to the subunit that binds Ecm29 (Rpt5, light blue in Fig. 1a).

### C-terminal tail of α7 subunit is important for Ecm29 binding to the proteasome

**Figure 2:**
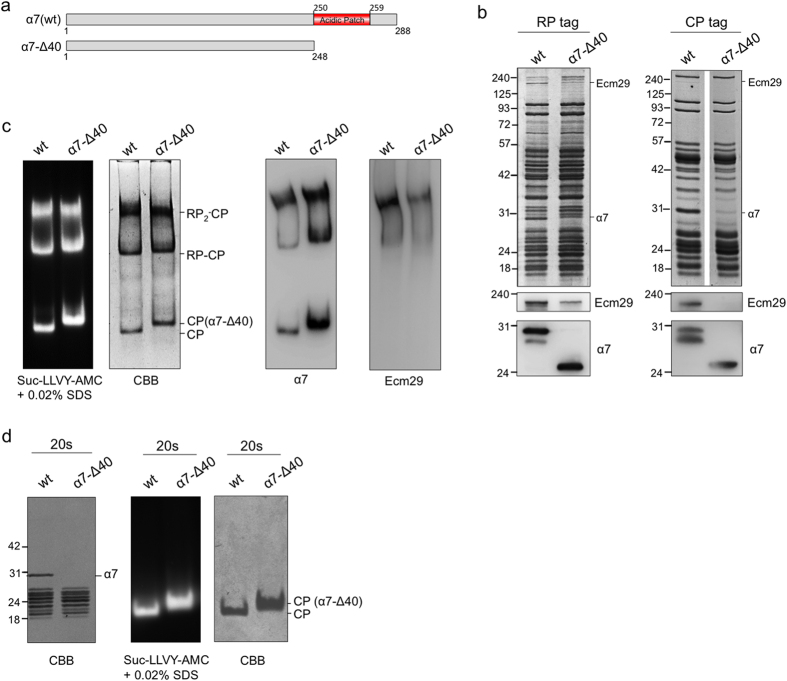
The C-terminal tail of the α7 is required for the binding of Ecm29 to the proteasome. **(a)** Schematic representation of wild type α7 displaying the acidic patch (residues D250 to D259) and the α7 with C-terminal truncation (α*7*-Δ*40*) that removes all amino acids beyond N248. **(b)** Proteasomes were purified from wild type and α*7*-Δ*40* cells using either an RP tag (Rpn11- TEV-ProA; left panel) or CP tag (Pre1-TEV-ProA; right panel) and analyzed on SDS-PAGE as well as by immunoblotting using antibodies against Ecm29 and α7. **(c)** RP-tag derived proteasome purifications from **(b)** were resolved on native gel and stained using an in-gel activity assay with LLVY-AMC as substrate in the presence of 0.02% SDS. Gel was coomassie stained or transferred to pvdf membrane to probe for the presence of Ecm29 and α7. **(d)** CP from wild type and α*7*-Δ*40* cells was resolved on SDS-PAGE and stained with CBB (left panel). Same samples were also resolved on native gel, followed by in-gel LLVY-AMC activity assay and CBB staining. Difference in electrophoretic migration of CP from α*7*-Δ*40* does not results from an enrichment in Blm10 and is likely due to the difference in charge between α*7* and α*7*-Δ*40*.

To test if the α7 C-terminal tail is important for Ecm29-proteasome interaction, we deleted the last 40 amino acid residues from the α7 subunit (Fig. 2a). These are residues that were not resolved in the crystal structure of the CP^30^. Strains with this α7 truncation were viable and we refer to the truncation as α*7*-Δ*40* from here onwards. To purify proteasomes we introduced this truncation into strains that either contained the protein A-tagged RP subunit Rpn11 or protein A-tagged CP subunit Pre1 (β4)^13^.

Compared with wild type proteasomes, proteasomes purified from these strains showed a similar pattern on Coomassie Brilliant Blue (CBB) stained SDS-PAGE gels (Fig. 2b). Immunoblotting for α7 showed a lower molecular weight for α7 upon truncation, consistent with the expected reduction in molecular weight from 31.5 kD to 27.3 kD. Purified proteasomes also showed a strongly reduced level of Ecm29 upon truncation of α7 (Fig. 2b). As is evident from the protein staining, proteasome preparations purified using the CP-tag have generally reduced levels of RP co-purified and increased amounts of CP as compared to RP-based proteasome purifications. This tag-induced difference likely explains the lower levels of Ecm29 in CP-tag proteasome purifications as compared to RP-tag preparations. However, there is consistently a clear reduction of Ecm29 levels upon deletion of the α7 tail.

Native gel analyses of proteasome complexes purified from wild type and α*7-*Δ*40* strains also showed reduced levels of Ecm29 in the α*7*-Δ*40* background. The small amount of Ecm29 that is still bound to proteasomes binds to the same complexes as has been observed under wild type conditions (Fig. 2c). Additionally, we observed a slower migrating form of CP in the α*7*-Δ*40* samples. A similar slower migration of CP is observed when CP associates with the CP interacting protein Blm10^32^. However, we did not observe increased levels of Blm10 in α*7*-Δ*40* proteasomes compared to wild type proteasomes (Fig. 2b). The α7 truncation contains 16 acidic residues and the calculated isoelectric point (pI) of the protein changes from 5.0 to 7.7, thus instead of other proteins binding to CP, the altered migration might be a consequence of the difference in charge between wild type and α*7*-Δ*40* CP. To test this we purified CP from wild type as well as from α*7*-Δ*40* (Fig. 2d). The high salt washes required to purify CP also removed any Blm10 that might be bound to the CP, as is apparent from the absence of ~240 kDa bands in CP preparations (Fig. 2d). Even in the absence of Blm10, α*7*-Δ*40* derived CP showed a retarded migration compared to CP with full-length α7. Thus, the change in migration results from the truncation and is most likely due to the loss of negatively charged amino acid residues. In sum, our data show that truncation of the α7 tail led to reduced binding of Ecm29 to proteasome.

### Effect of α7 C-terminal tail truncation *in vivo*

**Figure 3:**
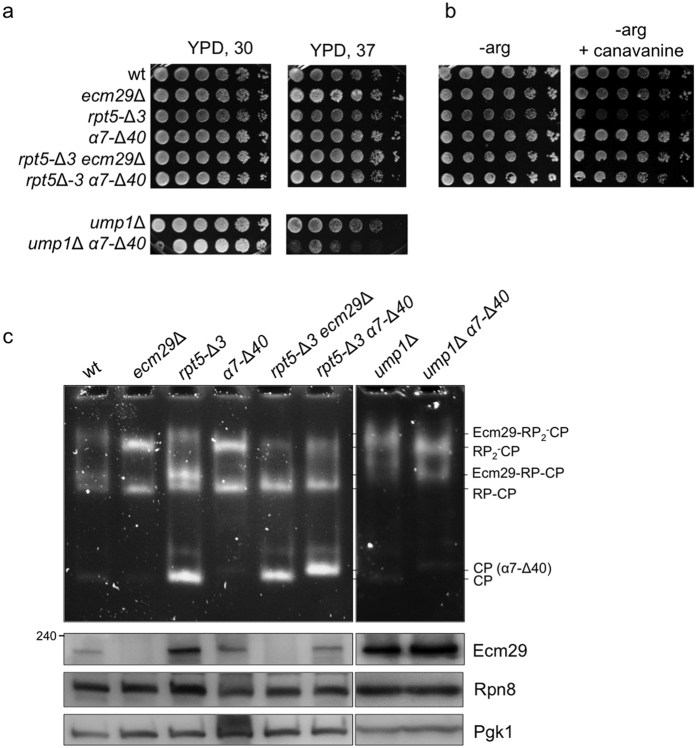
The α7 C-terminal tail is important for Ecm29 proteasomal association *in vivo*. (**a**,**b**) Indicated strains were serially diluted (four-fold) and **(a)** spotted onto YPD medium and grown at the indicated temperatures for 3 days or **(b)** spotted on SD plates lacking arginine with or without canvanine and grown at 30 °C for 5 to 6 days. The α*7*-Δ*40* truncation resemble a deletion of ECM29 in the different backgrounds. **(c)** Equal protein amount of total cell lysates from the indicated strains were analyzed on 3.5% nondenaturing gel and stained using the LLVY-AMC activity assay (upper panel). Protein extracts were also resolved on SDS-PAGE, followed by immuno blotting to determine the levels Ecm29 and Rpn8. Immunoblot against Pgk1 was used as a loading control.

Next, in order to determine the physiological importance of the α7 truncation *in vivo,* we analyzed cells for two phenotypes typical for proteasome mutants: sensitivity to heat stress at 37 °C and canavanine sensitivity. Both of these conditions are thought to introduce protein misfolding stress due to the high temperature or the incorporation of the arginine analog canvanine. Unlike several proteasome mutants or mutants with defects in proteasome assembly, the α7 truncation did not cause increased sensitivity to these conditions (Fig. 3a,b). This suggests there are no major defects in proteasome function upon deletion of the α7C-terminal tail. Similarly, the deletion of Ecm29 by itself does not cause temperature sensitivity or canavavine sensitivity.

Next, we tested if the truncation can rescue or cause synergistic effects in backgrounds where this has been observed for the deletion of Ecm29. Here, we predict that strains with the α7C-terminal truncation should, at least in part, mimic phenotypes that resulted from a deletion of *ECM29* in sensitized backgrounds, because α*7*-Δ*40* results in reduced binding of Ecm29 to proteasome. We have previously characterized the *rpt5-*Δ*3* mutant where the last three amino acids of the Rpt5 subunit are absent. In this strain proteasomes are enriched in Ecm29^23^. The Rpt5 residues that are missing in *rpt5-*Δ*3* strains are involved in the docking of RP onto CP^33^,^34^,^35^,^36^. The *rpt5-*Δ*3* strain shows canavanine sensitivity resulting from the cumulative effect of reduced proteasome activity caused by the mutant form of Rpt5 as well as the inhibition by Ecm29^14^. The canavinine sensitivity can be rescued by a deletion of *ECM29*. When we introduced the α7 tail truncation in *rpt5-*Δ*3* strains, the canavanine sensitivity was also rescued. Thus, the truncation of the α7 tail phenotypically mimics the deletion of *ECM29*.

The deletion of the assembly chaperone *UMP1* results in an enrichment of Ecm29 on proteasomes and the deletion of Ecm29 in an *ump1*Δ background causes increased sensitivity at 37 °C^24^. The α7C-terminal truncation in *ump1*Δ causes increased sensitivity at 37 °C as well (Fig. 3a), indicating the deletion of the 40 amino acids from the α7 causes a phenotype similar to the deletion of Ecm29 in this background as well. The deletion of Ecm29 or the α7C-terminal truncation does, however, not rescue the canavanine sensitivity of *ump1*Δ cells (Supplementary Figure 1). This might not be surprising considering the important role of Ump1 plays in assembly of the CP. In sum, the α7 truncation phenotypically mimics a deletion of Ecm29 in strains where *ecm29*Δ changes the phenotype of the strain (*rpt5-*Δ*3* and *ump1*Δ), consistent with our *in vitro* observations that the α7 tail is important for the binding of Ecm29 to proteasomes.

Next, we biochemically characterized proteasome complexes formed in the mutant strain background. Cell lysates were resolved on native gels followed by activity assays using the fluorogenic peptide LLVY-AMC (Fig. 3c). Singly and doubly capped proteasomes can associate with Ecm29. This association causes a slower migration on native gel^13^,^15^. Blm10 binding can also cause a slower migration, but only for singly capped proteasomes. However, our native gel analyses in the presence or absence of Blm10 indicates that the Blm10-containing species do not play a major role in the assays discussed below (Supplementary Figs 2 and 3). *Rpt5-*Δ*3* cells are highly enriched in Ecm29 as compared to wild type cells. Consequently, *rpt5-*Δ*3* proteasomes show a notably slower migrating species on the native gel, caused by association with Ecm29 as it is absent from *ecm29*Δ cells (Fig. 3c compare lane 3 and 5). Consistent with our phenotypic data, truncations of α7 in the *rpt5-*Δ*3* background caused a migration of the proteasomes on native gels similar to that resulting from the deletion of *ECM29*. This indicates a reduced recruitment of Ecm29 to α*7*-Δ*40 rpt5-*Δ*3* proteasomes.

To analyze the level of Ecm29 present in the different strains we ran the same samples on SDS-PAGE and conducted immuno blot analyses using an antibody that can recognize the endogenous Ecm29 protein (Fig. 3c lower panel). As observed previously, the *rpt5-*Δ*3* strain shows increased levels of Ecm29 in total cell lysate compared to wild type. Furthermore, truncation of α7 in this background reduces the levels of Ecm29 back to levels similar to wild type (Fig. 3c, compare lane 1, 3, and 6). Ecm29 has been reported to be degraded by proteasomes^24^,^25^, which might suggest that a loss of interaction with proteasomes increases Ecm29 turnover. However, other reports have suggested Ecm29 is stable and increased levels on proteasomes result from higher rates of transcription^17^. This would suggest the α7 tail truncation results in a lower level of Ecm29 expression in α*7*-Δ*40* mutant strains. In either case, the effect of the truncation of α7 seems to be background dependent, as in both the *ump1*Δ and the *ump1*Δ α*7*-Δ*40*, Ecm29 levels are increased as compared to wild type.

**Figure 4:**
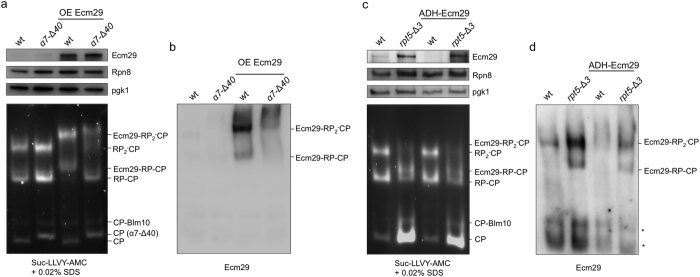
Exchange of Ecm29 promoter to eliminate differential transcription of Ecm29. **(a,b)** The endogenous promoter of Ecm29 was replaced with the GPD promoter. This exchange causes a strong overexpression of Ecm29 that drives binding of Ecm29 to all most all wild type proteasomes^14^,^17^, as is seen in the immunoblot of total lysates. Native gel analyses of total lysate can visualize presence of Ecm29 on singly and doubly capped proteasomes because the presence of Ecm29 causes a migrational shift. The presence was also confirmed by immunoblotting the native gel and probing with an anti-Ecm29 antibody. Even under conditions of strong overexpression the α*7*-Δ*40* strain shows strongly reduced levels of Ecm29 on singly and doubly capped proteasomes. **(c,d)** The endogenous promoter of Ecm29 was replaced with the ADH promoter resulting in weak expression of Ecm29 in wild type as well as in *rpt5-*Δ*3* strain background. Analyses were identical as for (**a,b**). With weak promoter the behavior of Ecm29 in wildtype and mutant strains is very similar to cells with the endogenous promoter, suggesting the Rpn4-dependent transcriptional regulation of Ecm29 is not essential for the enrichment of Ecm29 on proteasomes.

To eliminate complications in our analysis as a result of differences in transcriptional regulation of *ECM29*, we exchanged the promoter of *ECM29* with the *GPD* promoter. For wild type cells this has been previously shown to increase the levels of Ecm29 in the cell and result in stoichiometric amounts of Ecm29 on proteasomes^14^,^17^ (Fig. 4a,b). Under these conditions we see high levels of Ecm29 in the cell lysate of both wild type and α*7*-Δ*40* cells. Native gel analyses, however, clearly shows a reduced level of Ecm29 on 26S proteasomes from the α*7*-Δ*40* strains, with singly capped proteasomes having hardly any Ecm29 associated with the proteasome and strongly reduced amount of doubly capped proteasomes associated with Ecm29. Thus, even under conditions of very strong overexpression of Ecm29 the truncation of α7 causes reduced binding of Ecm29 to proteasomes. Since Ecm29 binds to Rpt5 in addition to α7, the lost of α7 binding probably results in a substantially reduced affinity of Ecm29 for proteasomes, but may not invoke a complete loss of binding. This can explain some limited binding of Ecm29 to proteasomes in the α*7*-Δ*40* background.

The ability to drive Ecm29 association with the proteasome by increasing the levels of Ecm29 in the cell suggests that Ecm29 association with proteasomes is driven by the transcriptional regulation of *ECM29* in the cell. However, we have previously shown that Ecm29 shows better binding to mutant proteasomes as compared to wild type proteasomes^14^. Thus, an alternative explanation for the observation of Ecm29 on wild type proteasomes is that a lower affinity of Ecm29 for wild type proteasomes can be obscured by strong overexpression of Ecm29. Ecm29 levels resulting from GPD promoter are for example substantially higher than any increase resulting from upregulation of the endogenous locus through the stabilization of Rpn4. Thus, analyses from GPD promoter driven Ecm29 overexpression results are non-physiological. Therefore, we created strains where Ecm29 expression is not driven by the very strong GPD promoter, but by the much weaker ADH promoter (Fig. 4c,d). Here, we still eliminate the Rpn4 feedback loop by replacing the endogenous promoter, but now have a much lower transcription level of *ECM29*. Consistent with this, there is less Ecm29 in the cells as compared to wildtype cells (Fig. 4c). That notwithstanding, the level of Ecm29 in the cell and on the proteasome dramatically increase in *rpt5-*Δ*3* cells with the ADH promoter. These cells very much resemble our observations made in *rpt5-*Δ*3* cells which have the endogenous promoter. Thus, Rpn4 driven transcriptional upregulation is not required to achieve increased association of Ecm29 with mutant proteasomes.

### α7 phosphorylation is required for Ecm29 interaction with CP

**Figure 5:**
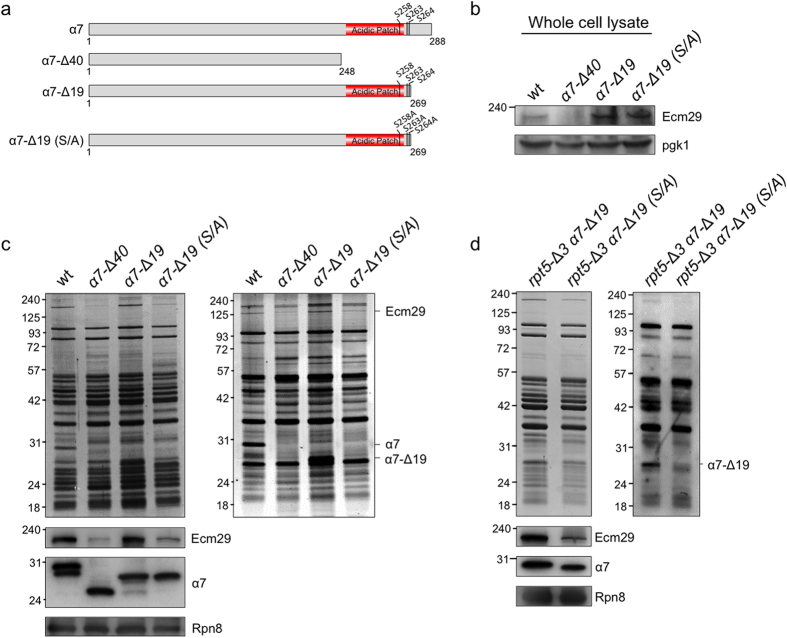
Binding of Ecm29 to proteasomes depends on the phosphorylation of the C-terminal tail of α7. **(a)** Schematic overview of α7 truncations and mutations used. A shorter truncation of the α7 C-terminal tail, α*7*-Δ*19* (1–269), was created. This truncation retains the acidic patch as well as three serine phosphorylation sites (S258, S263 and S264). Mutation of these phosphorylation sites to alanine in this background resulted in the α*7*-Δ*19* (S/A) strain. **(b)** Ecm29 levels in indicated strains was analyzed by immunoblotting of whole cell lysates using the anti-Ecm29 antibody and an anti-Pgk1 antibody as loading control. (**c,d**) Proteasome complexes were purified from the indicated strains and equal amount of purified proteasomes were analyzed on the SDS-PAGE and Coomassie Blue stained or used for immunoblotting to determine level of Ecm29. Immublots against α7 were used to confirm truncations and immunoblots agains Rpn8 served as loading control. To determine phosphorylation state, samples resolved by SDS-PAGE were stained with Pro-Q Diamond Phosphoprotein Gel Staining assay (right panels).

Seven phosphorylation sites have been identified in yeast α7^37^,^38^,^39^,^40^,^41^,^42^,^43^. Interestingly, five of these sites, S258, S263, S264, T278 and T279, are present in the tail of the α7 that we eliminated. From these, S258, S263, and S264 have been identified multiple times and also in studies specifically focused on phosphorylation of the proteasome^37^,^39^,^40^,^43^. The phosphorylation of α7 has been observed in humans as well, suggesting there is a conserved function for this modification^44^,^45^,^46^. CK2 (formerly known as casein kinase 2) has been proposed to be responsible for phosphorylation of the serine residues present with the α7C-terminal tail^37^,^47^. CK2 generally phosphorylates serine residues in an acidic environment^48^, which is provided by the acid patch present in the α7 tail. Furthermore, the phosphorylation by CK2 is often constitutive. This is consistent with the observation that yeast as well as human α7 is present almost exclusively in the phosphorylated form^43^. To test if α7 is phosphorylated under our conditions, we purified proteasomes and resolved preparations on SDS-PAGE. Gels were stained with CBB and analyzed using the in-gel pro-Q diamond phosphostain. These analyses show a band corresponding to the size of α7 that is strongly phosphorylated. Consistent with our assignment of α7, this band disappears in the phospho-stain analyses when analyzing proteasomes from the α*7*-Δ*40* strain (Fig. 5c, right panel). Since no band of lower molecular weight appeared, our data show that α7 is mainly phosphorylated in the C-terminal fragment, as recently suggested^43^. To test if T278 and T289 in α7 are essential for the ability of Ecm29 to bind proteasomes, we eliminated these phosphorylation sites by making a truncation that removes only 19 residues instead of 40 from α7, keeping the acidic patch and three serine phosphorylation sites intact, namely S258, S263, and S264. We refer to this mutant as α*7*-Δ*19* (Fig. 5a). Ecm29 is detectable in these strains (Fig. 5b). Proteasomes purified from an α*7*-Δ*19* strain showed a band of the expected size on a phospho-stain gel, indicating that the remaining phosphorylation sites are still phosphorylated (Fig. 5c). The levels of Ecm29, as determined by immunobotting, are similar to those of Ecm29 found associated with wild type proteasome (Fig. 5c). Thus, Ecm29 binding is mediated by the fragment of the α7 tail that contains the acidic patch and the serine phosphorylation sites. To test the importance of phosphorylation of these serine residues, we introduced serine to alanine mutations into the α*7*-Δ*19* background. The strains with all three serine residues mutated to alanine (S258A, S263A, S264A) is referred to as α*7*-Δ*19* (S/A). Proteasomes purified from α*7*-Δ*19* (S/A) cells showed no detectable phosphorylation of α7 in our phospho-stained gel assay (Fig. 5c). Interestingly, these proteasomes showed dramatic reduction in the amount of Ecm29, having levels similar to those on α*7*-Δ*40* derived proteasomes. Consistent with an important role for serine phosphorylation in the binding, mutations of the serine residues to aspartate retained normal levels of Ecm29 on the proteasomes (supplementary Fig. 4). Thus, the binding of Ecm29 to proteasomes requires the phosphorylation at serine residues in the tail of α7.

It is currently unclear if the binding of Ecm29 to mutant proteasomes relies on fundamentally different proteasome binding sites or if Ecm29 utilizes the same binding sites for binding to wild type and mutant proteasomes. To test this for the phsophorylated α7 tail, we introduced the α*7*-Δ*19* (S/A) mutant in the *rpt5-*Δ*3* background. Upon purification of proteasomes we observed that mutation of the serines in the *rpt5-*Δ3 background still leads to reduced levels of Ecm29 on proteasomes. Thus, the recruitment of Ecm29 to faulty proteasomes relies on the ability of Ecm29 to bind to CP as well as RP.

## Discussion

Many sites of post-translational modification have been identified on the proteasome^49^. Some functions for proteasome phosphorylation have been described, like enhanced degradation of substrates^50^,^51^,^52^,^53^, regulation of proteasome activity in particular compartments^54^, or regulation of proteasome assembly or stability^55^. While the mechanisms are often unclear, the phosphorylated residues of proteasome subunits have been proposed to (directly) regulate the proteasomal ATPase activity, state of the CP gate, or the proteolytic active sites. Here, we report that the phosphorylated tail of α7 is important for binding of the proteasome-associated factor Ecm29. While it has been suggested that phosphorylation of the α7 tail might leads to a conformational change in RP or CP^49^, we think this is unlikely based on our data and recent published work^43^ and propose a direct binding of Ecm29 to the α7 tail. Irrespectively, this is the first example where the association of a proteasome interacting proteins depends on a phosphorylated proteasome subunit.

Often phosphorylation is a transient modification that has a local effect for a limited period of time and affects only a subset of a particular protein. Both in humans and yeast, however, the phosphorylation of the proteasome α7 subunit appears to be constitutive, readily detectable, and present on >95% of the α7 subunits^43^. Ecm29, on the other hand, is only found on a subpopulation of proteasomes, suggesting that the phosphorylation of α7 is not the distinguishing factor that determines which proteasomes bind Ecm29. Instead of being a trigger that creates a binding site for Ecm29, α7 phosphorylation appears to provide a pre-requisite for binding. Consistent with this model, the kinase that likely phosphorylates α7, CK2, is a constitutive kinase with many substrates in the cell^37^,^47^,^48^. CK2 phosphorylation has for example also been proposed to be a prerequisite for the UBC3 binding to the F-box receptor β-TrCP^56^. To identify functions of α7 phosphorylation it will be crucial to identify a cellular condition where the phosphorylation is reduced. Immature CP could be one such condition, considering Ecm29 is normally only found on mature assembled proteasomes. However, in immature CP α7 is already phosphorylated (Supplementary Figure 5a). An alternative to identify functions of α7 phosphorylation is the identification of a phosphatase responsible for the removal of the phospho-groups can provide valuable insight. An important role for a phosphatase in regulating proteasome function has been reported previously, with UBLCP1 regulating nuclear proteasomes^54^, but no yeast ortholog of this phosphatase exists.

The phosphorylation of the α7 tail is important for association of Ecm29 with proteasomes, but it remains unclear if that is the only function of the phosphorylated tail of α7. Previous studies have reported that in humans the phosphorylation of α7 is important for regulating the stability of the CP-RP interactions^46^, but this was not linked to Ecm29. Ecm29-dependent stabilization of CP-RP is particularly striking in the absence of nucleotide, where yeast proteasomes would dissociate otherwise. In the absence of Ecm29 a similar stabilization can be achieved by treating proteasomes with proteasome inhibitors^15^. However, this stabilization does not depend on the tail of α7 (Supplementary Figure 5b). Thus, it remains to be determined if α7 can stabilize CP-RP directly as well as through Ecm29.

The α7 tail might also serve to bind or recruit other factors besides Ecm29. Several proteins have been reported to bind to α7, including several ubiquitin-independent proteasome substrates^57^,^58^. The role of the C-terminal tail here remains to be clarified.

As predicted during the initial identification of Ecm29 as a proteasome associated protein in 2001, and validated herein, Ecm29 binds to both RP and CP^13^,^14^. The identification of these sites provides an important clue towards the mechanism of Ecm29 recruitment to specific proteasomes subpopulations. Nevertheless, we do not fully understand why Ecm29 is present in substoichiometric amounts on wild type proteasomes, but is highly enriched on a variety of proteasome mutants. Ecm29 does not appear to compensate for defects by stabilizing proteasomes, but instead to specifically bind and inhibit mutant proteasomes^14^,^17^,^23^. Phenotypically, the effect of deleting Ecm29 is rather pleiotropic, suggesting it has multiple functions in the cell or becomes a dominant negative factor under certain conditions. Either way, the mechanisms responsible for enrichment of Ecm29 on mutant proteasomes are still poorly understood.

Three models have been put forward to explain enrichment of Ecm29 on mutant proteasomes: first, Rpn4-dependent enrichment^17^. In this model, reduced proteasome activity as a result of a mutation in proteasome subunits leads to the accumulation of the unstable transcription factor Rpn4^59^. Rpn4 recognizes the PACE element found in the promoter of Ecm29 as well as many proteasome subunit^60^. As a result, both proteasome subunits and Ecm29 are upregulated. However, Ecm29 upregulation is stronger, thereby causing a relative increase in Ecm29 as compared to proteasomes^17^. Since we observed Ecm29 enrichment on mutant proteasomes in strains where the endogenous promoter was replaced with the ADH promoter (Fig. 4c), it is clear that this mechanism is not solely responsible for proteasomal enrichment of Ecm29. That said, it probably is an important contributing factor and other type of proteasome mutants might rely more heavily on this mechanism.

The second model proposes a high affinity of Ecm29 for mutant proteasomes^14^. This model is consistent with our observation that strong overexpression of Ecm29 can compensate for a weaker affinity and drive proteasomal binding of Ecm29 to normal proteasomes. However, weaker promoters, like the ADH promoter, prevent this and thus result in an almost exclusive accumulation of Ecm29 on mutant proteasomes. Although we lack the molecular insight into a mechanism that would allow for such a difference in affinity, it might involve differences in the relative position of the Ecm29 binding sites on CP relative to RP.

**Figure 6:**
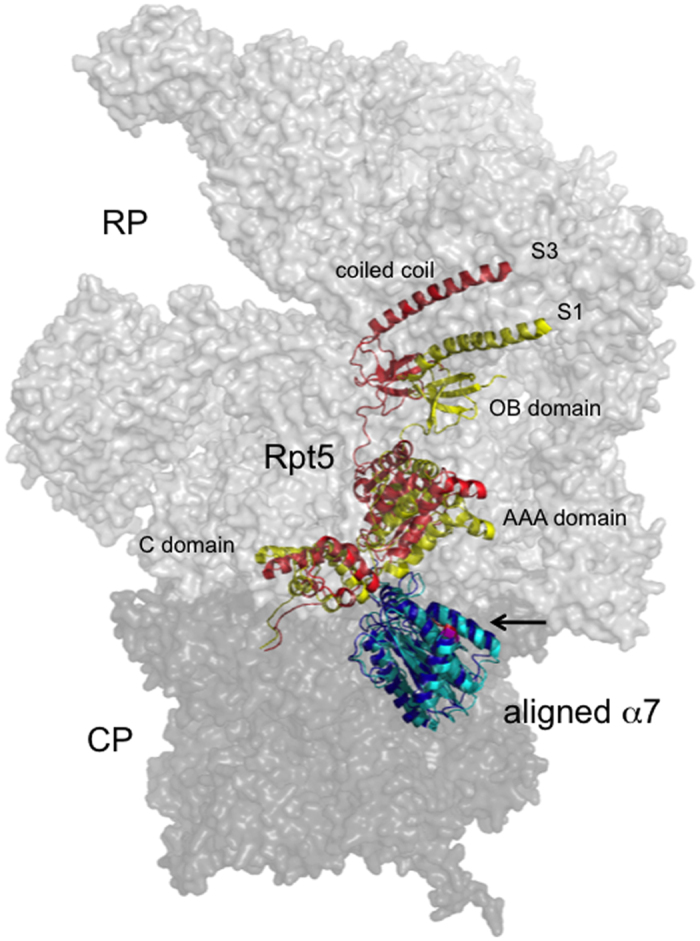
Structural changes in the 26 S proteasome at the Ecm29 binding region. Three different conformations of the mature proteasome^61^ (4CR2, 4CR3, and CR4) were aligned in Pymol using the α7 domain. The intermediate state (S2, 4CR3) is depicted in transparent gray, for the other states, S1 (4CR2) and S3 (4CR4) only the α7 and Rpt5 subunits are depicted in color. The red residues in α7 show the last three resolved residues (245-247; EIN), the phosphorylated residues that are important for Ecm29 binding extend from here, but are not resolved. Ecm29 also binds to Rpt5, but the precise site has not been mapped.

The interaction between CP and RP has some flexibility as the tails of the Rpt5 has been found to interact with CP at different positions^26^. Furthermore, analyses of the different cryoEM structures of the proteasome has revealed differences in the CP-RP interface that are probably linked to the nucleotide status of the ATPases^61^,^62^. Using, three states with reported PDB structures^61^ (S1, a ground state; S2, an intermediate state; S3 an activated, ATPγS-bound, state) we aligned the α7 subunit to reveal difference in relative position of Rpt5 compared to α7. We noticed substantial differences in the Rpt5 position (Fig. 6). For example, the distance between α7 I246 and Rpt5 D209 changes from 32.4 Å in the S1 state to 42.3 Å in the S3 state. This likely indicates the relative position of the two binding surfaces Ecm29 uses to interact with the proteasome changes depending on the proteasome conformation. Such changes could explain differences in binding affinity of Ecm29 for particular proteasome conformations. Consistent with this model, it has been proposed that Ecm29 might bind or trap proteasomes in the S1 state^3^, thereby inhibiting proteasomal ATPase activity. Enrichment of Ecm29 on proteasome mutants could then be a result of increased occupation of a ground state by these mutant proteasomes. However, the majority of wildtype proteasomes, at least in neurons, appears to be in a ground state^63^. So, there might be as of yet uncharacterized states of the proteasome that Ecm29 has high affinity for. This idea is supported by observations that Ecm29 bound proteasomes have a closed gate, something not observed in the S1 state^14^,^17^,^23^. Furthermore, proteasomes depleted for nucleotides, a condition that leads to RP-CP dissociation and thus is different from S1, show increased Ecm29 binding and Ecm29-dependent stabilization of RP-CP^14^,^15^. Since, many mutants that accumulate Ecm29 are linked to deficiencies in RP-CP interactions, direct or indirectly, these could accumulate a different proteasome state^12^,^20^,^21^,^31^.

Alternatively, a higher affinity of Ecm29 to proteasomes could be induced by local changes in the structure, for example if a mutation of the Rpt5 subunit changes the shape directly or through the nucleotide occupancy of Rpt5. However, the enrichment of Ecm29 on *Rpt5-*Δ*3* proteasomes still depends on the ability of Ecm29 to bind to the phosphorylated α7 tail.

The third model is based on differences in Ecm29 degradation. This model proposes that Ecm29 is degraded by the proteasome. Hence, any defects in proteasome function result in stabilization of Ecm29 and a subsequent enrichment of Ecm29 on proteasomes. While this model is simple and elegant, our observations that Ecm29 inhibits proteasomes suggests Ecm29 would prevent its own degradation. Tagged versions of Ecm29 have been reported to be degraded^24^,^25^ and degradations rates could also be influenced by internal disordered regions are more or less accessible for engagement by the proteasome, like has been shown for other substrates^64^. Less availability of such regions when Ecm29 binds mutant proteasomes could dramatically change protein half-life. However, endogenous Ecm29 has been reported to be stable^17^, leaving little support for this model.

In sum, our data show that phospohorylation of the tail of α7 is a prerequisite for the binding of Ecm29 to proteasomes. Since Ecm29, the extended α7 tail, and the CK2 phosphorylation are found in human as well, this binding mechanism is likely conserved form yeast to humans. While this suggests that the interaction is regulated by phosphorylation, the conditions leading to the dephosphorylation of α7 and thus dynamic regulation of Ecm29 binding through phosphorylation remain to be identified.

## Methods

### Yeast techniques, plasmids and reagents

Yeast strains used are summarized in Table 1. Genomic manipulation of yeast was done using a PCR based approach^65^,^66^. Upon transformation of yeast, successful integration was confirmed by positive PCR for integration and negative PCR for wild type. To make α*7*-Δ*40*, primer pRL207: 5′-TGC TAC AGG AAG CTA TCG ATT TTG CCC AAA AAG AAA TTA ACT GAG GCG CGC CAC TTC T-3′ and pRL66: 5′-TCA ACT CTT TGG TTC TTC TTA ACG TAT TAT CAG AAT GTC ATC GAT GAA TTC GAG CTC G-3′ were used. α*7*-Δ*19* that contains the acidic patch and phosphorylation sites, primers pRL 248: 5′-TGA CAG TGA TAA CGT CAT GTC CAG TGA TGA TGA AAAT GCT TGA GGC GCG CCA CTT CT-3′ and pRL66, for α*7*-Δ*19* (S/A) that replaces three Serine to Alanine (S258A, S263A and S264A) from α7 tail pRL319: 5′-ATC GAT TTT GCC CAA AAA GAA ATT AAC GGC GAT GAT GAC GAG GAC GAA GAT GAC GCG GAT AAC GTC ATG GCG GCC GAT GAT GAA AAT GCT TGA GGC GCG CCA CTT CT-3′ and pRL 66 were used. To make α*7*-Δ*19* (S/D) that represents the phospho mimic (S258D, S263D and S264D), pRL320: 5′-ATC GAT TTT GCC CAA AAA GAA ATT AAC GGC GAT GAT GAC GAG GAC GAAG ATG ACG ACG ATA ACG TCA TGG ATG ACG ATG ATG AAA ATG CTT GAG GCG CGC CAC TTC T-3′ and pRL66 were used. All truncations and point mutations were confirmed by PCR followed by sequencing. To delete C-terminal last three amino acids from Rpt5 subunit, pRLS2-Rpt5: 5′-AAT ATG TAG ATA TGT GAA TGG CGG CTT GAT AAA TCA AAA TAT TAT TAT TTA TCG ATG AAT TCG AGC TCG-3′ and pRLS3-Rpt5-Δ3: 5′-TCG TTG AGG GTA TAA GTG AAG TTC AAG CAA GAA AAT CGA AAT CGG TAT CCT AGG GCG CGC CAG ATC TGT T-3′ were used. The templates used for the PCR reactions indicated above were pFA61-3HA-kanMX6 (KAN) and pYM24 (HYGRO), primers were designed to eliminate the 3-HA tags.

Ecm29 promoter changes were done by using pRL194: 5′-TCT CCA CGA GCT GTT TTT CTT TCG CTT CGT CAG AAG AAA TGG A TC CGG AAT GGT GAT GGT GAT GGT GGT GCA TCG ATG AAT TCT CTG TCG-3′ and pfEcm29 Ntag s1: 5′-CAA TAA TTA TAG AAA AGT TTC TAT TTC ACC ACG AAC AAC ATT CGT ACG CTG CAG GTC GAC-3′ with pYM-N15 to add GPD overexpression promoter. To add ADH weak expression promoter to Ecm29, pYM-N7 was used with pRL194 and pfEcm29-Ntag-s1. Integrations, truncations and mutations were confirmed by PCR and sequencing.

### Antibodies used for immunoblots

Ecm29 and Rpn8 were detected using polyclonal antibody (generous gift by Dan Finley, Harvard Medical School, Boston, MA). Anti-α7 monoclonal antibody and anti-Blm10 poly clonal antibody were purchased from Enzo Life Sciences and anti-Pgk1 monoclonal antibody was purchased from Invitrogen. Peroxidase-conjugated secondary antibodies were purchased from Jackson Immunoresearch Laboratories.

### Proteasome purification

Specific yeast strains were grown overnight in 2 to 3 liter of YPD media (final A_600_ ~ 10.0). Cells were collected and washed with H_2_O. Pellets were resuspended in 1.5 pellet volume of lysis buffer (50 mM Tris [pH8.0], 5 mM MgCl_2_, 1 mM EDTA, and 1 mM ATP) and lyzed using a French Press at 1200 Psi. Lysates were cleared by centrifugation (10,000 g; 30 minutes) and the supernatant was filtered through cheesecloth. Concentration of cell lysate was measured using NanoDrop 2000 Spectrophotometer using the Protein A_280_ program and cell lysate equivalent to 50 μg of protein was boiled with 1X SDS-PAGE sample buffer for 5 minutes at 96 °C. Next, samples were resolved on 11% SDS-PAGE. For proteasome purification cell lysate were filtered through cheesecloth prior to incubation with IgG beads (MP Biomedical; 0.75 ml resin bed volume per 25 gram of cell pellet). After 1 hour of incubation (rotating at 4 °C), IgG beads were collected in an Econo Column (Bio-Rad) and washed with 50 bed volumes of ice-cold wash buffer (50 mM Tris [pH 7.5], 5 mM MgCl_2_, 50 mM NaCl, 1 mM EDTA, 1 mM ATP). Next, material was washed with 20 bed volumes of cleavage buffer (50 mM Tris [pH 7.5], 5 mM MgCl_2_, 1 mM DTT, and 1 mM ATP). Bead-bound proteasome complex was eluted by incubation with His-Tev protease (Invitrogen). The protease was removed by incubation with talon resin (Goldbio) prior to concentrating the proteasome complexes using 100 kDa concentrator (PALL Life Sciences). Purified proteasome were resolved on SDS-PAGE or on native gel as described previously^67^. To store samples, 5% final concentration glycerol was added and samples were stored at −80 °C.

For core particle purification proteasome bound IgG beads were incubated with 5 bed volumes of wash buffer containing 500 mM NaCl for 1 hr at 4 °C under constant rotation. After incubation, the beads were washed with 50 bed volumes of wash buffer with 500 mM NaCl. Elution and concentration of core particle complexes were as described above for proteasome complexes.

### Yeast phenotypes

Indicated strains were incubated in 3 ml of YPD for overnight at 30 °C. Next day, cells equivalent to 1.0 at OD_600_ were collected and washed with 100 μl sterile water. Cell pellet is then resuspended in 133 μl of sterile water and 4-fold serial dilutions were made in sterile water in 96 well plate. Diluted cells were then spotted on the indicated plates and incubated at indicated temperatures. Growth was monitored for 3 days on YPD plates and for 5–6 days on synthetic media plates.

### Fluorescent phospho- and Coomassie Blue staining

10 μg Proteasome complex purified from the indicated samples were mixed with the 6X loading buffer and boiled for 5 minutes at 95 °C. Boiled samples were cooled to room temperature and resolved on 11% SDS-PAGE gel. To detect the phosphorylated proteins, gel was subjected to fluorescent photo staining by using ProQ Diamond Phosphoprotein Gel Stain according to the manufacturer’s recommendations (Life Technologies) and scanned with a Typhoon-9410 imager from Amersham Biosciences (excitation at 532 nm and an emission filter of 560 nm longpass). After destaining, the gel was treated with Coomassie Blue and imaged using a Gbox image system (SynGene) with GeneSnap software.

### Apyrase assay

Proteasome purified from the indicated strains were inhibited using 200 nM of Epoxomicin at 30 °C for 30 minutes. Both inhibited and non-inhibited proteasome samples are then subjected to apyrase treatment for 45 minutes at 30 °C by adding apyrase (Sigma) at a final concentration of 20 mU/μl in buffer (50 mM Tris-HCl [pH 7.5], 5 mM MgCl_2_, 0.25 mM ATP). Samples are then analyzed on native gels followed by in-gel activity assay using LLVY-AMC as substarte. Next, protein resolved in gel were transfered to pvdf membrane as described previously and used for immunoblotting^14^.

## Additional Information

**How to cite this article**: Wani, P. S. *et al*. Phosphorylation of the C-terminal tail of proteasome subunit α7 is required for binding of the proteasome quality control factor Ecm29. *Sci. Rep.* **6**, 27873; doi: 10.1038/srep27873 (2016).

## Supplementary Material

Supplementary Information

## Figures and Tables

**Table 1:** Strains used in this study.

Strain	Genotype	Figure	Ref.
sUB61	MAT α lys2-801 leu2-3, 2-112 ura3-52 his3-Δ200 trp1-1	3a–c, 4a–d,	68
sDL135	MAT α pre1::PRE1-TEV-ProA (HIS3)	2b, 2d	13 (a)
sDL133	MAT α rpn11::RPN11-TEV-ProA (HIS3)	2b–c, 5b–d	69
sMK141	MAT α ecm29::TRP	3a–c,	14
sJR 211	MAT α ecm29::TRP rpn11::RPN11-TEVProA (HIS3)	6a	(b)
sJR544	MAT α ecm29::TRP rpt5::RPT5Δ3 (HYG)	3a–c	23
sJR552	MAT A rpt5::RPT5Δ3 (HYG) rpn11::RPN11-TEVProA (HIS3)		23
sJR556	MAT A rpt5::RPT5Δ3 (HYG)	3a–c, 4c–d,	23
sJR768	MAT α pre10::PRE10Δ40 (KAN)	3a–c, 4a–b	(a,b)
sJR770	MAT α pre10::PRE10Δ40 (KAN) pre1::PRE1-TEVProA (HIS3)	2b, 2d	(a,b)
sJR805	MAT A pre10::PRE10Δ40 (KAN) rpt5::RPT5Δ3 (HYG)	3a–c,	(a,b)
sJR810	MAT α pre10::PRE10Δ40 (KAN) rpn11::RPN11-TEVPro (HIS3)	2b–c, 5b–d	(a,b)
sJR816	MAT A ecm29:: pADH-H_7_-MYC-ECM29 (NAT)	4c–d	(b)
sJR820	MAT A ecm29:: pADH-H_7_-MYC-ECM29 (NAT) rpt5::RPT5Δ3 (HYG)	4c–d	(b)
sJR822	MAT α ecm29::TRP pre10::PRE10Δ40 (KAN) rpn11::RPN11-TEVPro (HIS3)	6a	(a,b)
sJR839	MAT A ecm29:: pGPD-H_7_-MYC-ECM29 (NAT)	4a–b	(b)
sJR840	MAT A pre10::PRE10Δ40 (KAN) ecm29:: pGPD-H_7_-MYC-ECM29 (NAT)	4a–b	(a,b)
sJR856	MAT A ump1Δ pre10::PRE10Δ40 (HIS3)	3a–c, 4a–d	(b)*
sJR884	MAT α pre10::PRE10Δ19(KAN) rpn11::RPN11-TEVPro (HIS3)	5b–c	(a,b)
sJR887	MAT α pre10::PRE10Δ19(S/A) (KAN) rpn11::RPN11-TEVPro (HIS3)	5b–c	(a,b)
sJR888	MAT α pre10::PRE10Δ19(S/D) (KAN) rpn11::RPN11-TEVPro (HIS3)		(a,b)
sJR911	MAT α pre10::PRE10Δ19(S/A) (KAN) rpt5::RPT5Δ3 (HYG) rpn11::RPN11-TEVProA (HIS3)	5d	(b)
sJR912	MAT α pre10::PRE10Δ19 (KAN) rpt5::RPT5Δ3 (HYG) rpn11::RPN11-TEVProA (HIS3)	5d	(a,b)
*ump1*Δ	MAT A ump1Δ	3a–c, 4a–d	70*
Ump1-TAP	MAT A ump1::UMP1-CBP-TEV-ZZ- (His3MX6)	6b	*

^*^In the BY4741 background (*MAT* α *his3*Δ*1 leu2*Δ*0 met15*Δ*0 ura3*Δ*0*).

^a^*PRE1* encodes for the proteasome subunit β4 and *PRE10* for α7.

^b^This study.

All strains have background genotype (*lys2-801 leu2-3, 2-112 ura3-52 his3-Δ200 trp1-1*), with the exception of the ump1 manipulated strains indicated with*.
