# Supplementary material for: Phosphorylation of the C-terminal tail of proteasome subunit α7 is required for binding of the proteasome quality control factor Ecm29
Source: Sci Rep. 2016 Jun 15;6:27873. doi: 10.1038/srep27873 (PMC4908598; doi:10.1038/srep27873)
Supplement: Supplementary Information [file srep27873-s1.pdf]

SUPPLEMENTARY INFORMATION

Phosphorylation of the C-terminal tail of proteasome subunit α7 is required for binding of the proteasome quality control factor Ecm29

Prashant S. Wani, Anjana Suppahia, Xavier Capalla, Alex Ondracek, and Jeroen Roelofs

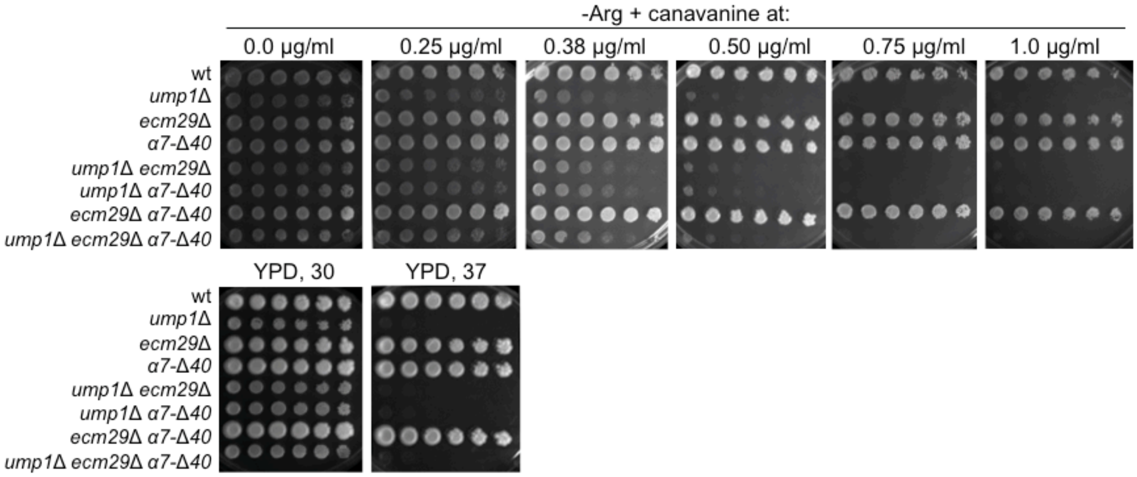

Supplementary Figure 1. Reduced binding of Ecm29 to proteasomes does not rescue the phenotype of *ump1*Δ. *ump1*Δ cells have increased levels of Ecm29, however, upon deletion of Ecm29 or reduced binding of Ecm29 (*α7*-Δ*40*) they remain sensitive to canavanine. This is probably indicative of functions Ump1 has in CP assembly that are affected by Ecm29. Note that the increased temperature sensitivity observed in the BY4741 background (Fig. 3a main text) was not observed in the DF5 background used for all strains here. Strains used from top to bottom Sub61, sJR946, sJR950, sJR936, sJR943, sJR937, sJR941, sJR939.

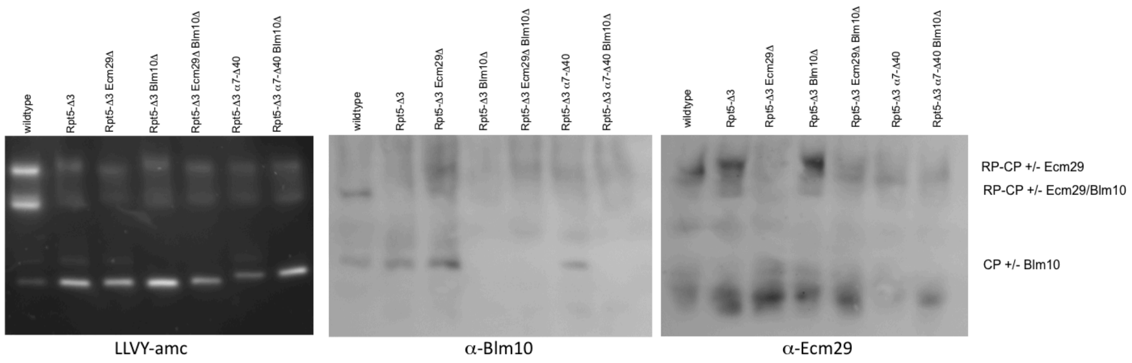

Supplementary Figure 2. Ecm29 binds to RP-CP in *Rpt5-Δ3*. In wildtype type cells Blm10 is the major component responsible for slower migration of RP-CP, however Ecm29 can also cause a slower migration of the RP-CP species. To determine the contribution of Ecm29 and Blm10 towards the slower migrating RP-CP species in the *Rpt5-Δ3* background, indicated strains were lysed and protein complexes were separated on native gel. After in gel peptidase activity assay (using LLVY-amc in the presence of SDS) samples were transferred to pvdf and immunoblotted for Blm10 and Ecm29. Lysate from *Rpt5-Δ3* cells showed increased amounts in slower migrating RP_2-CP an RP-CP. The shifted RP_2-CP species is solely due to Ecm29 (compare lanes 2,3, and 4 top band). In *Rpt5-Δ3* the increase in slower migrating RP-CP was largely due to Ecm29, because the slower migrating RP-CP from *Rpt5-Δ3* showed less Blm10 on immunoblots (the distinct band in lane 1 middle panel is reduced in lane 3; see also supplementary figure 3). Furthermore, upon deletion of Blm10 the slower migrating RP-CP band remains present in *Rpt5-Δ3* and is positive for Ecm29 (compare lane 2 and 4). The increased presence of Ecm29 on singly and double capped proteasomes in the *Rpt5-Δ3* is reduced upon deletion of the α7 tail (lanes 6 and 7). Strains used have DF5 background and were from left to right SUB61, sJR951, sJR953, sJR955, sJR957, sJR949, and sJR958.

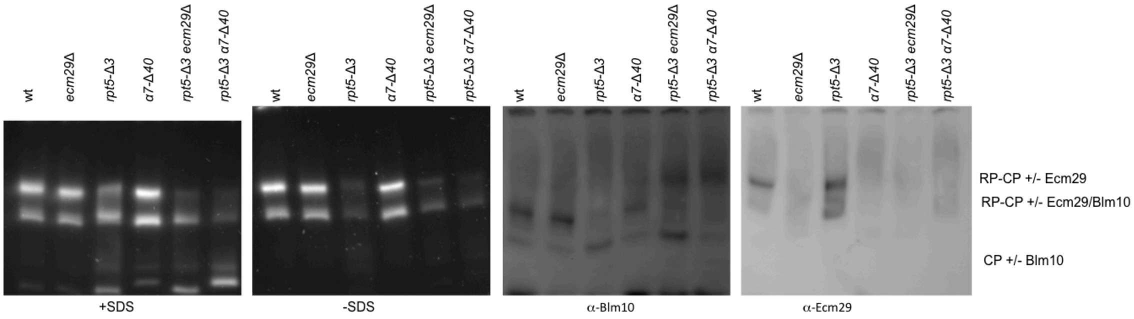

Supplementary Figure 3. Analyses complementary to Figure 3. Lysates of indicated strains were separated on native gel, assayed for in gel peptidase activity in the presence and absence of SDS (0.02%). Next, samples were transferred to pvdf membrane and immunoblotted for Blm10 and Ecm29. Ecm29 inhibits proteasomes by reducing ATPase activity and by closing the gate. In the left two panels, lane 3 shows the effect Ecm29 has on CP gating, as these Ecm29 containing proteasomes show little peptidase activity in the absence of SDS, but are activated upon artificially opening the gate with SDS. The difference between lane 3 and 6 on the immunoblot for Ecm29 illlustrates the importance of the α7 tail for Ecm29 association with proteasomes. Strains used have DF5 background and were from left to right Sub61, sMK141, sJR556, sJR768, sJR544, and sJR805.

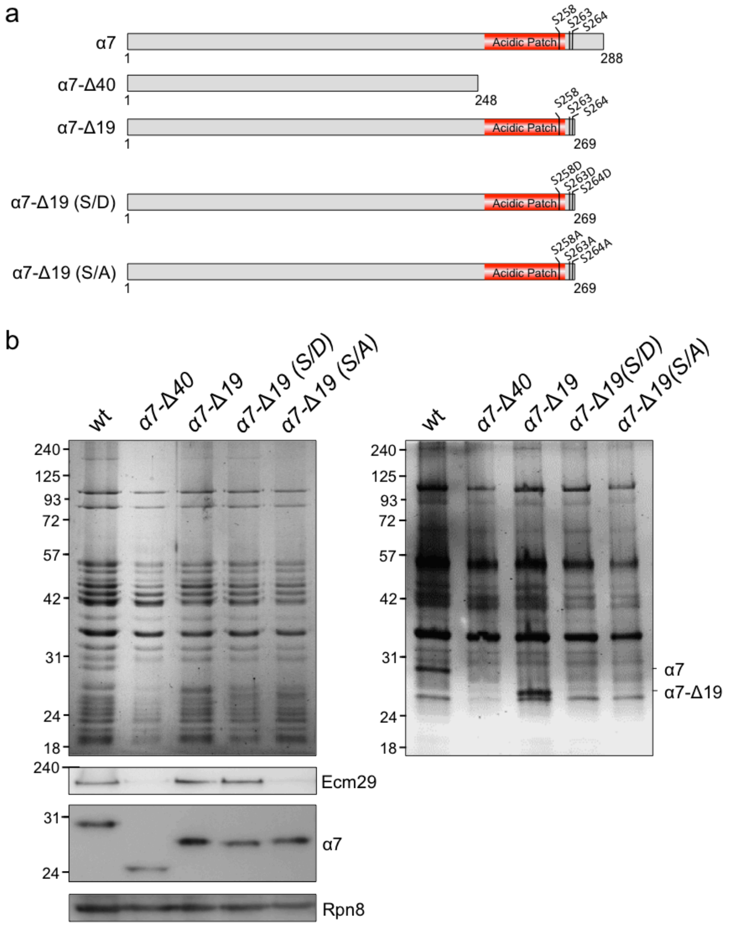

Supplementary Figure 4. Phosphomimetic mutations of the serine residues in the C-terminal tail of α7 accommodate Ecm29 binding. (a) Schematic overview of α7 truncations and mutations used. A shorter truncation of the α7 C-terminal tail, *α7-Δ19* (1-269), was created. This truncation retains the acidic patch as well as three serine phosphorylation sites (S258, S263 and S264). Mutation of these phosphorylation sites to alanine in this background resulted in the *α7-Δ19* (S/A) strain. Mutation of these phosphorylation sites to aspartates in this background resulted in the *α7-Δ19* (S/D) strain (**b**) Proteasome complexes were purified from the indicated strains and equal amount of purified proteasomes were analyzed on the SDS-PAGE and Coomassie Blue stained or used for immunoblotting to determine level of Ecm29. Immublots against α7 were used to confirm truncations and immunoblots against Rpn8 served as loading control. To determine phosphorylation state, samples resolved by SDS-PAGE were stained with Pro-Q Diamond phosphoprotein gel staining (right panels). Mutation of the serine residues results in loss of the phosphorylation as can be observed from the proQ diamond phospho-stain. The phosphomimetic mutations (S/D) maintain Ecm29 binding, while the (S/A) show strongly reduced Ecm29 binding. Strains used from left to right sDL133, sJR810, sJR844, sJR887, and sJR888.

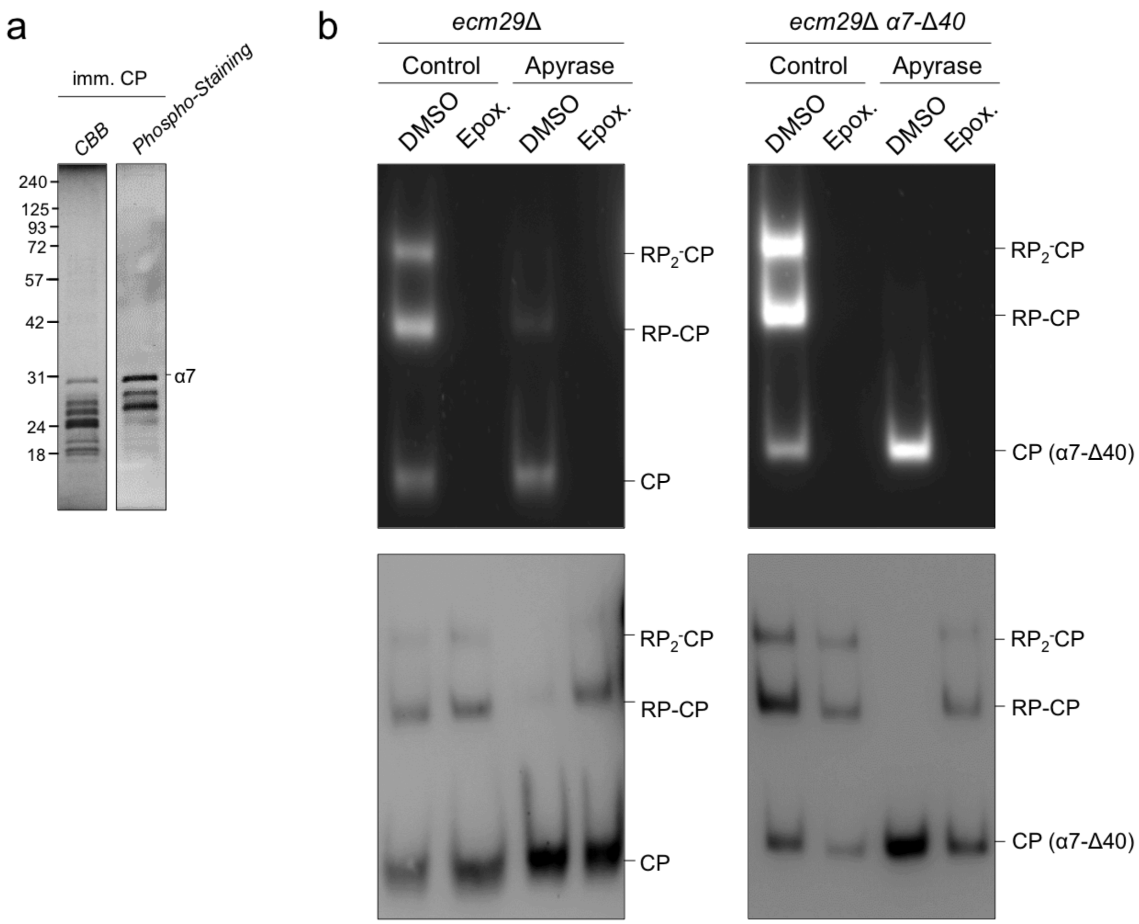

Supplementary Figure 5. Function not reglated by the C-terminal tail of α7. (**a**) Immature proteasomes were purified and analyzed by SDS-PAGE followed by Coomassie Blue staining and phosphostaining using ProQ-Diamond phospho-stain. α7 in immature CP is phosphorylated, indicating that the absence of Ecm29 from immature proteasomes is not due to the lack of α7 phosphorylation. (**b**) Proteasomes purified from *ecm29Δ* cells or *ecm29Δ* α*7-Δ40* were treated with proteasome inhibitor or DMSO prior to incubation with or without apyrase. Apyrase converts ATP and ADP into AMP, resulting in a dissociation between CP and RP [13] (compare lane 1 and 3). The treatment with the proteasome inhibitor epoxymycin prevents this independent of the presence or absence of the α*7* C-terminal tail.
